# Immature Gastric Teratoma: A Rare Tumour

**Published:** 2010-12-01

**Authors:** Muhammad Sharif, Bilal Mirza, Lubna Ijaz, Shahid Iqbal, Afzal Sheikh

**Affiliations:** Department of Pediatric Surgery, The Children's Hospital and the Institute of Child Health Lahore, Pakistan

**Keywords:** Gastric teratoma, Immature teratoma, Infant

## Abstract

Gastric teratomas are very rare tumours in children. They usually present with a palpable mass in the upper abdomen. We report a case of gastric teratoma in one and half month old male infant who presented with a palpable mass in abdomen, extending from epigastrium to the pelvis. Ultrasound of abdomen revealed a huge mass with solid and cystic components. CT scan delineated calcifications in the mass. The preoperative diagnosis was a teratoma but not specifically gastric one. The mass was excised completely with seromuscular layer of the stomach wall. The histopathology confirmed it to be grade-3 immature gastric teratoma. The rarity of the origin of teratoma in addition to its immature variety prompted us to report the case.

## INTRODUCTION

Gastric teratoma is an extremely rare tumour in pediatric age, accounting for less than I% of all teratomas diagnosed in this age group. To date, only less than 100 cases have been reported in literature [1,2].

Immature gastric teratomas are even rarer [3]. This report describes a case of immature gastric teratoma in an infant 

## CASE REPORT

One and half month old male infant presented with mass abdomen, noted by parents, for a week that has progressed gradually. There was nothing significant on general physical examination. Abdomen was distended with a palpable mass that extended from epigastrium down to the pelvis. 

All the laboratory parameters were within normal limits except alpha fetoprotein (AFP) which was 110 ng/ml. Ultrasound abdomen showed a huge mass in midline having solid and cystic components. Abdominal radiograph depicted a mass effect in the central abdomen displacing the bowel loops and stomach. CT scan abdomen revealed a mass with different densities and calcifications in central part of the tumor (Fig. 1). Preoperative diagnosis was abdominal teratoma.

**Figure:**
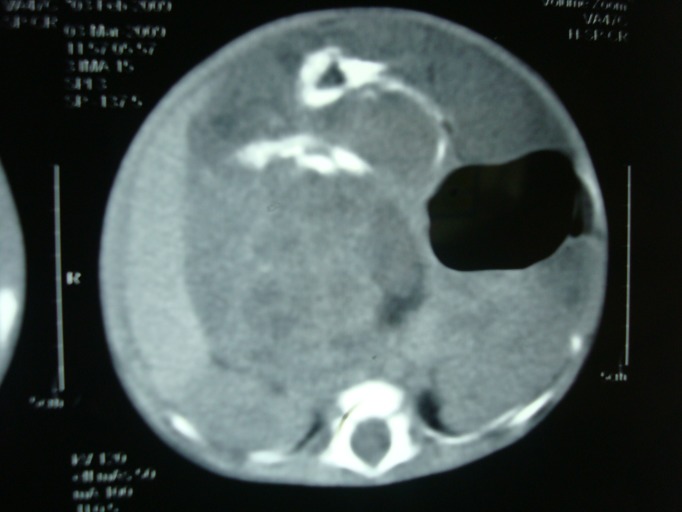
Figure 1: CT scan showing a mass with solid and cystic components along with internal calcifications.

Patient was explored through a right supra-umbilical transverse incision. A huge mass arising from the posterior wall of stomach, extending down to the pelvis found (Fig. 2). 

**Figure:**
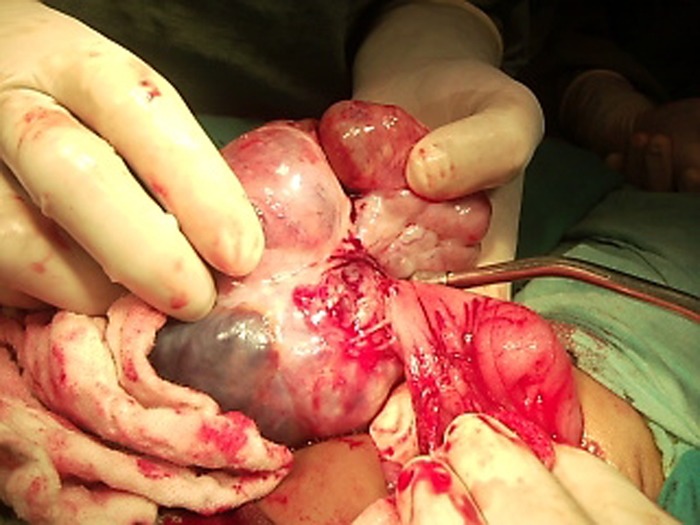
Figure 2: Showing dissection of the teratoma from posterior wall of the stomach.

It was excised in-toto along with some portion of seromuscular layer of stomach while sparing the gastric mucosa. The gastric seromuscular defect was repaired. Specimens were sent for histopathology. The report came as a neoplasm derived from all three germ layers. It was comprised of immature glial tissue (at various sites), fibrocollagenous tissue and adipose tissue, multiple variable sized cysts lined by respiratory epithelium, and cartilage and bone. These features were consistent with immature teratoma grade 3. Biopsy from margins of origin was free of tumour. Postoperative course was uneventful. At one month follow up the AFP was decreased to 10 ng/ml. Patient was followed up to 6 months and later did not report back.

## DISCUSSION

The frequently occurring teratomas in pediatric patients comprise of sacrococcygeal teratoma followed by those originating from mediastinum, gonads, retroperitoneal region and so on. Gastric teratomas are extremely rare tumours and cases are frequently reported in infancy and neonatal period (> 90%), however, there have been reports of this tumour in older children as well [1-3].

First case of gastric teratoma was reported in 1922 by Eustermann et al. Margret et al had reported a case of gastric teratoma in a day old newborn. Gamanagatti et al had published a case report of gastric teratoma in a 2 year old boy [3-5].

The site of origin of gastric teratoma is variable though most of the cases have been reported to arise from the greater curvature and posterior wall of the stomach as found in the index case, however, other sites such as lesser curvature have also been documented [6].

The clinical features depend upon the site of origin, size, and endogastric component. Exogastric growths are common (58-70%) in contrast to endogastric growths (30%). The usual features are abdominal distension and mass followed by vomiting, constipation, and respiratory distress. In case of intramural extension the patients may present with hemetemesis, pain abdomen, melena, vomiting, and rarely with gastric perforation [6,7]. Our patient belonged to the exogastric variety.

Abdominal radiograph usually delineates a mass effect that displaces the bowel gas shadows to a side. In about 50% cases abdominal radiographs can also pick the calcifications and bone densities which are hall mark of intra-abdominal teratomas. CT scan is the modality of choice. When combined with intravenous and oral contrasts, they can detect the origin of the tumour, its relation with gastrointestinal tract and major blood vessels, presence of bones and calcifications, and tumour extent and presence of metastasis. Other modalities like barium meal and gastroscopy has a limited role in the diagnosis of gastric teratoma [6-8].

The monitoring of AFP and beta-HCG reflect the treatment response after excision and may be of significant value where chemotherapy is recommended (immature variety) [6]. In our case the preoperative AFP level was abruptly reduced by ten times after a month of excision. 

The histopathology confirms the diagnosis and states about the maturity of the teratoma. A grading system, based on histopathological findings, divides the gastric teratoma in two main varieties viz. mature teratoma (grade 0) and immature teratoma (grade 1, 2, 3). In mature teratoma, mature and well differentiated tissues belonging to all the three germinal layers, is present. In case of immature teratoma, immature neuroectodermal tissue is usually found along with other germinal layer structures. In grade 1 immature teratoma the immature neuroectodermal tissue is confined to one site in a slide, where as in grade 2 and 3 the immature tissue is usually found in less than 4 and more than 4 fields per slide, respectively [6-8]. Our case falls in grade 3 immature teratoma on the basis of presence of immature neuroglial element at multiple sites. 

Most of the gastric teratomas are considered to be benign but malignancy in gastric teratoma and malignant gastric teratomas have also been reported. Only few cases of immature teratomas have been published in literature. Malignant potential increases with higher histopathological grades. Many authors depicted no recurrence after complete excision of the immature gastric teratoma even in grade 2 and 3; and there had not been any use of postoperative chemotherapy or radiotherapy, however in a case where the AFP start rising after few months of complete excision of teratoma, chemotherapy is added [6-10]. The recommended treatment of any gastric teratoma is complete surgical excision (with tumor free margins) and close surveillance. We adopted same approach. Recurrence in immature teratoma in a neonate has been reported [10]. In our case patient was lost to follow up after six months thus final outcome can not be commented upon.

To summarize, gastric teratomas are extremely rare tumours and rare still are their immature variety. Complete surgical excision with tumor free margins and long term follow up are the standard management principles to be adopted.

## Footnotes

**Source of Support:** Nil

**Conflict of Interest:** None declared
